# Demographic associations for autoantibodies in disease-free individuals of a European population

**DOI:** 10.1038/srep44846

**Published:** 2017-03-28

**Authors:** Kadri Haller-Kikkatalo, Kristi Alnek, Andres Metspalu, Evelin Mihailov, Kaja Metsküla, Kalle Kisand, Heti Pisarev, Andres Salumets, Raivo Uibo

**Affiliations:** 1 Institute of Bio- and Translational Medicine, Department of Immunology, University of Tartu, Ravila 19, Tartu 50411, Estonia; 2 Competence Center on Health Technologies, Tiigi 61b, Tartu 50410, Estonia; 3 Institute of Clinical Medicine, Department of Obstetrics and Gynecology, University of Tartu, L. Puusepa 8, Tartu 51014, Estonia; 4 Estonian Genome Center, University of Tartu, Riia 23b, Tartu 51010, Estonia; 5 Institute of Molecular and Cell biology, University of Tartu, Riia 23, Tartu 51010, Estonia; 6 Department of Public Health, University of Tartu, Ravila 19, Tartu 50411, Estonia; 7 Institute of Bio- and Translational Medicine, Department of Biomedicine, University of Tartu, Ravila 19, Tartu 50411, Estonia; 8 Department of Obstetrics and Gynecology, University of Helsinki and Helsinki University Hospital, Helsinki, FI-00029 HUS, Finland

## Abstract

The presence of autoantibodies usually precedes autoimmune disease, but is sometimes considered an incidental finding with no clinical relevance. The prevalence of immune-mediated diseases was studied in a group of individuals from the Estonian Genome Project (n = 51,862), and 6 clinically significant autoantibodies were detected in a subgroup of 994 (auto)immune-mediated disease-free individuals. The overall prevalence of individuals with immune-mediated diseases in the primary cohort was 30.1%. Similarly, 23.6% of the participants in the disease-free subgroup were seropositive for at least one autoantibody. Several phenotypic parameters were associated with autoantibodies. The results suggest that (i) immune-mediated diseases are diagnosed in nearly one-third of a random European population, (ii) 6 common autoantibodies are detectable in almost one-third of individuals without diagnosed autoimmune diseases, (iii) tissue non-specific autoantibodies, especially at high levels, may reflect preclinical disease in symptom-free individuals, and (iv) the incidental positivity of anti-TPO in men with positive familial anamnesis of maternal autoimmune disease deserves further medical attention. These results encourage physicians to evaluate autoantibodies in addition to treating a variety of patient health complaints to detect autoimmune-mediated disease early.

Autoantibodies are immunoglobulins (Ig) produced by activated autoreactive B cells. The immune response towards self-antigens usually involves activation of both T and B cells, but the detection of autoantibodies in sera is technically simpler than detection of T-cell reactions. Therefore, autoantibodies can be used to guide clinical management of certain diseases. These markers of disease activity and severity help to define and classify diseases and can be used to predict and diagnose specific autoimmune diseases^1^.

Autoimmune diseases affect at least 5% of the population^1^, while the prevalence of diseases that involve immune reactions, including connective tissue diseases (CTD) and diseases with hypersensitivity reactions, is much higher^2^. In reality, the actual burden of various (auto)immune reactions in different populations is unknown.

Some autoantibodies are functional and are therefore considered clinically significant, while the others are bystanders in disease pathogenesis (or their function has not yet been discovered). For example, IgG-type autoantibodies to the 100 kDa membrane bound glycoprotein thyroid peroxidase (anti-TPO) interrupt the production of thyroid hormones and cause autoimmune hypothyroiditis^3^. In addition, anti-TPO IgGs have been detected in cases of Graves’ disease and postpartum thyroid dysfunction, but they have also been detected in control individuals without thyroid disease^1^. Therefore, anti-TPO represents an autoantibody with tissue-specificity and clinical significance unspecific to thyroiditis. On the contrary to tissue-specific autoantibodies which are produced against antigens expressed in single tissue, tissue non-specific antigens recognise antigens expressed ubiquitously or at least in several tissues. IgA-type autoantibodies against the 78 kDa tissue transglutaminase (anti-tTG) are highly specific to coeliac disease^4^, making them clinically significant but not tissue specific. Although tTG belongs to a family of multifunctional transglutaminases, in coeliac disease, the anti-tTG IgAs produced in the small-intestinal mucosa interrupt the conversion of a glutamine residue into glutamic acid during gluten digestion^5^.

Depending on the disease, time of testing, and the number and detection level of autoantibodies, the sensitivity of predicting autoimmune disease is rarely 100%^1^. In other words, there are always individuals who test positive for autoantibodies but have no clinical signs of autoimmune disease for years. Similarly, there are cases where autoimmune disease develops without prior clinical indication. Therefore, the interpretation of positive autoantibody tests can be challenging in diseases such as thyroiditis, as anti-thyroid autoantibodies may precede disease manifestation by two decades, and some individuals (10%) stay disease-free despite the presence of autoantibodies^6^. Data interpretation is further complicated in diseases with complex pathologies, such as CTD, an autoimmune-inflammatory disease^7^. Furthermore, autoantibodies may be produced temporarily to facilitate communication between immune cells and molecules or between immune cells and other tissues, particularly during immune challenges such as viral infections^8^.

The prevalence and relevance of autoantibodies in healthy individuals are poorly studied, and most data found in the literature are derived from assessing autoantibodies in patients with autoimmune diseases^1^,^4^. Two outstanding questions that remain unanswered are how often autoantibodies can be detected in clinically healthy individuals and whether the presence of autoantibodies predicts the future onset of autoimmune disease. A prospective follow-up survey of selected individuals would be the gold standard to study these questions^6^, but needs highly synchronized medical efforts for organizing such studies and significant financial resources.

In this study, we aimed to determine the prevalence of selected clinically significant autoantibodies in (auto)immune-mediated disease-free individuals and to carry out an association study to explain the existence of autoantibodies in these healthy individuals. Namely, the data from a population-based registry of 51,862 adults from the Estonian Genome Center at the University of Tartu, Estonia (EGCUT) was used to assess (auto)immune-mediated diseases in the general population of Estonia. The study group of healthy individuals representative of the population was randomly selected from that registry. Study individuals were tested for anti-TPO IgG and 5 tissue non-specific autoantibodies diagnostic of major (auto)immune-mediated diseases. The presence of autoantibodies was assessed in relation to phenotypic characteristics in disease-free individuals.

## Materials and Methods

### Study population

This cross-sectional study comprised 994 individuals who lacked autoimmune or other immune-mediated diseases. Participants were selected from the 51,862 individuals aged 18 years or older in the EGCUT biopank, which is a population-based biopank compiled from 2002 to 2014 (www.biobank.ee)^9^. All study protocols were conducted in accordance with the Estonian Gene Research Act.

The EGCUT cohort closely reflected the age, sex, and geographical distribution of the Estonian population^9^, and all subjects were recruited voluntarily and randomly by general practitioners and hospital physicians. A computer-assisted personal interview, which included personal, genealogical, educational and occupational history, and lifestyle data, was completed. Anthropometric measurements, blood pressure, and resting heart rate were measured, and venous blood was drawn during the visit. Medical history and current health status were recorded according to International Classification of Diseases -10 (ICD-10) codes. The individuals excluded from the study of diagnoses with (auto)immune-mediated diseases are listed in Supplementary Table S.1. Random selection of the individuals representative of the age and gender distribution in the Estonian population resulted in 1,000 individuals. However, following a secondary survey of medical records of these 1,000 individuals, an additional 6 individuals possessing at least one of the (auto)immune-mediated diseases were excluded from the study group. Thus, the total number of study participants was 994 (491 men and 503 women ranging from 18 to 86 years old). Phenotypic characteristics and ethylenediaminetetraacetic-acid-treated (EDTA) plasma samples from each individual were used. Each participant signed an informed consent prior to enrolment, and ethical approval for the study was obtained from the Ethics Review Committee on Human Research at the University of Tartu.

Phenotypic data, including socio-demographic data, smoking and alcohol consumption, medication use, parents’ diseases, and female reproductive health-related data were used. Socio-demographic data consisted of age at agreement (age at time of study), nationality, city or rural residence at birth, occupation, and body mass index (BMI). Medication data consisted of medications that were used on a regular basis for the last two months. Medications were classified according to the Anatomical Therapeutic Chemical Classification System level. A study participant was considered to be using a hazardous drug if he/she used a medication associated with drug-induced lupus as described in Chang *et al*.^10^.

### Antibody tests

All participants were tested for anti-TPO and 5 tissue non-specific autoantibodies - antinuclear antibodies (ANA), which were measured with CTD IgG screening test (anti-CTD), anti-tTG IgA and IgG, cyclic citrullinated peptide IgG (anti-CCP), and antibodies of all isotypes against glutamic acid decarboxylase with molecular weight of 65 kDa (GADA). Anti-CTD was further specified with the eight most common IgG-type autoantibodies: antibodies against double-stranded deoxyribonucleic acid (anti-dsDNA), Sjögren’s syndrome type A antigen (anti-SS-A/Ro) protein, Sjögren’s syndrome type B antigen (anti-SS-B/La) protein, centromere protein (anti-CENP), histidyl-tRNA synthetase (anti-Jo-1) protein, scleroderma-associated autoantigen of 70 kDa (anti-Scl-70) protein, Smith (anti-Sm) protein, and ribonucleoprotein U1 (anti-U1RNP) protein.

GADA were measured with commercial enzyme-linked immunosorbent assay (ELISA) kit (RSR Ltd., Cardiff, UK). Plasma from EDTA-vacutainers was treated with calcium^11^. According to the manufacturer’s instructions, values 5 U/ml or grater were considered positive.

Anti-TPO, anti-CCP, anti-tTG IgA and IgG, anti-CTD, and autoantibodies from CTD tests were analysed with a fully automated fluoro-enzyme immunoassay (FEIA) on an ImmunoCAP 100 (Phadia, Thermo Scientific Corporation, Vantaa, Finland). Positive cut-off values were chosen according to the manufacturer’s recommendations: ≥100 IU/ml for anti-TPO, ≥10 U/ml for anti-CCP, ≥10 U/ml for anti-tTG, and an autoantibody ratio ≥1.0 for anti-CTD. To specify anti-CTD, all positive and grey zone (0.7–1.0) test results were reassessed with the eight most common ANAs: anti-dsDNA, anti-SS-A/Ro, anti-SS-B/La, anti-CENP, anti-Jo-1, anti-Scl-70, anti-Sm, and anti-U1RNP IgG. Anti-dsDNA values 15 IU/ml or more were considered positive, while the cut-off value for the remaining 7 autoantibodies was set to 10 U/ml, according to the manufacturer’s instructions.

### Statistical analyses

The Welch Two Sample *t*-test and Proportion test with continuity correction were used to compare the reported phenotypic characteristics and prevalence of autoantibodies between men and women, and *p* values < 0.05 were considered significant. Multiple logistic regression analyses adjusted for age and stratified by gender were used to find associations between the presence of autoantibodies and phenotypic data. For association analyses, autoantibodies were considered binomial variables for logistic regression – (i) the presence of anti-TPO IgG as a tissue-specific autoantibody and (ii) the presence of at least one of the 5 tissue non-specific autoantibodies: GADA, anti-CCP IgG, anti-tTG IgG and IgA, or anti-CTD IgG. The age at time of study was considered a continuous variable, and a categorical variable of age groups was formed based on the values of the first and third quantiles and median age and compared with the youngest group (18–26 years). Adjusted odds ratios (adORs) were calculated, and corrected *p* values < 0.05 were considered statistically significant, while corrected *p* values > 0.05 and <0.1 were considered tendencies for a significant association. The R3.1.0 Language and Environment was used for statistical analyses.

The data set supporting the results of this article is included within the article and it’s Supplementary Table.

## Results

### Health parameters of the study population

According to data registered by EGCUT by May 2014, approximately 30% of the adult population in Estonia has been diagnosed with some type of (auto)immune-mediated disease (Supplementary Table S.1). As expected, women were diagnosed more frequently than men (32.1% vs. 26.4%), except in the case of insulin-dependent (type 1) diabetes, which occurred more frequently among men (0.5%) than women (0.3%, Table 1). Coeliac disease was diagnosed very rarely in the population (0.04%), and the frequency was not different in men and women. More important in the context of the current study is the fact that 2/3 of the adult population was registered healthy and free from (auto)immune-mediated diseases at the time of recruitment into the EGCUT registry and formed the basis for the selection of our study group – 994 (auto)immune disease-free individuals representative of the entire Estonian population of adults in the distribution of age and gender.

The patient characteristics and health parameters of the study group (994 individuals) are provided in Table 2. The mean age of the participants was 39.9 years. The study population also contained approximately equal numbers of men and women who originated from the city or the countryside. The prevalence of employment and student status was similar between genders, but more men were retired, and more women were unemployed due to reasons other than retirement. Smoking was greater among men. Men also started smoking at younger ages. If men did consume alcohol, they did it more frequently than women. Seventy-three percent of women reported that they had been pregnant, and 68% of all women had at least one child. More women in the study population were normal weight than men, who were more often overweight. The prevalence of both extremes, underweight (BMI <18.5 kg/m^2^) and obesity (BMI ≥30 kg/m^2^), were similar between genders. Regular medication use in the most recent 2 months was also similar between men and women. The prevalence of individuals taking drug(s) included in the Anatomical Therapeutic Chemical Classification System or capable of inducing autoimmune diseases^10^, ranged from 1.3–16.0%, and did not differ between men and women. More women than men (16.7% vs. 11.4%) reported that their mothers had at least one (auto)immune-mediated disease and men reported more frequently same diseases in their father’s (Table 2).

### Prevalence of and associations with autoantibodies

The most prevalent autoantibodies detected in this study were GADA (8.8%), anti-TPO IgG (7.2%), and anti-CTD (4.8%). The total prevalence of at least one autoantibody was 23.6% (Table 3), while the co-existence of two or more autoantibodies was found in 2% of participants (95% confidence interval (CI): 0.0–8.0). The prevalence of detected autoantibodies in comparison of data from literature is presented in Table 4.

Autoantibodies were divided into two groups for further association analyses according to specificity. Anti-TPO IgG was classified as a tissue-specific autoantibody, and the tissue non-specific autoantibodies included GADA, anti-CCP IgG, anti-tTG IgG and IgA, and anti-CTD IgG. After adjusting for age and stratifying by gender, linear and logistic regression analyses revealed the phenotypic characteristics that were significantly associated with autoantibodies (Table 5). These characteristics were used as adjustments for association analyses of autoantibodies.

**Figure 1:**
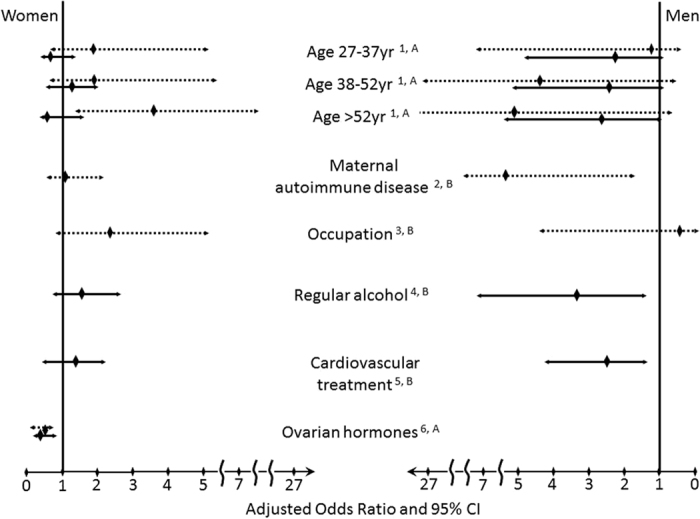
Risk factors for autoantibodies detected in men and women. Autoantibodies against thyroid peroxidase (anti-TPO, dashed line) and any tissue non-specific autoantibody (solid line) groups are shown separately. The tissue non-specific autoantibody group comprised individuals who tested positive for at least one of the 5 tested tissue non-specific autoantibodies. ^1^age groups were formed according to the values of 1^st^ and 3^rd^ quantiles and the median age compared to the youngest group (18–26 years); ^2^the presence of maternal autoimmune disease compared to individuals without maternal autoimmune disease; ^3^individuals with an occupational status of unemployed but being covered by pension insurance (includes retired individuals and unemployed individuals with insurance for disability) compared to employed persons; ^4^seldom but regular alcohol consumption (once a month) compared to non-consumers; ^5^the need for cardiovascular treatment on a regular basis at least 2 months prior to the study; ^6^women with active ovarian hormones includes women who menstruate and women with primary or secondary amenorrhea who receive hormone replacement therapy compared to women in menopause. Association values are given as odds ratios considered for multiple comparisons from multivariate logistic regression models stratified by gender and adjusted for the following confounders selected from Table 5: ^A^ age groups; ^B^ age groups (or ovarian hormonal activity in women), maternal autoimmune disease, occupational status, alcohol consumption, cardiovascular treatment. CI –confidence interval.

Multivariate regression model simultaneously controlled for all significant parameters from Table 5 was used to calculate corrected *p* value from multiple comparisons (corrected *p* value < 0.05 was considered statistically significant). These risk factors with the adjusted ORs are shown in Fig. 1. The presence of anti-TPO IgG was assessed in the gender-stratified logistic regression model adjusted for age, maternal autoimmune disease, and occupational status (significant phenotypic parameters for anti-TPO, Table 5). The analyses revealed that the presence of anti-TPO autoantibodies in men was independently associated with (i) the presence of maternal autoimmune disease (adOR = 5.51, *p* = 0.001 compared to men whose mothers were not suffering from autoimmune diseases), (ii) older age (adOR = 1.05, *p* = 0.008 for one year of age, data not shown in Fig. 1), and (iii) tended to be associated with occupational status of being student or serviceman (adOR = 3.86, p = 0.065). The same model for women showed an increased risk of anti-TPO for age group older than 52 years (adOR = 3.54, *p* = 0.014 for ages >52 years compared to the youngest women of 18–26 years of age) and risk for anti-TPO was not increased at younger age groups. The presence of at least one tissue non-specific autoantibody in men was assessed in association with the phenotypic parameters of age, frequency of alcohol consumption, and need for cardiovascular treatment (significant phenotypic characteristics for current autoantibodies, Table 5). This model revealed that the likelihood of having one tissue non-specific autoantibody in men tended to be increased by (i) age (adORs for age groups 27–37 years, 38–52 years, and older than 52 years were 2.15, *p* = 0.072; 2.21, *p* = 0.059; and 2.31, *p = *0.054, respectively, compared to the youngest group, 18–26 years of age), (ii) the odds were higher if a man consumed alcohol regularly (adOR = 3.33, *p* = 0.004 compared to non-consumers), and required cardiovascular treatment (adOR = 2.31, *p* = 0.011). The age adjusted and gender stratified model for women revealed that women were less likely positive for tissue non-specific autoantibodies in case of active ovarian hormones (adOR = 0.45, *p* = 0.036 for women who were either menstruating or were receiving HRT compared to women in menopause and not receiving HRT) (Fig. 1).

The most prevalent autoantibody GADA was detected in 8.8% of individuals of disease-free population, if the cut-off value for GADA positivity was selected >5 U/ml. However, a proportion of individuals were tested GADA greater than 10, 30 or even 50 U/ml (Table 6). Multivariate logistic regression analyses revealed that women with positive anti-CTD IgG test were associated with increased chance for GADA >10 U/ml and >30 U/ml (adORs 4.27, *p* = 0.017 and 5.65, *p* = 0.047, respectively) when the model was controlled by the ovarian hormonal activity. At the same time, active ovarian hormonal status decreased the likelihood of GADA >10 U/ml and >30 U/ml (adORs 0.34, *p* = 0.027, and 0.17, *p* = 0.038, respectively). The results were similar, if models were adjusted by the age of older and younger than 45 years instead of hormonal activity (data not shown). The age 45 was selected to unify data presentation between men and women. These results suggest that finding of either high level of GADA or anti-CTD IgG in disease-free women may reflect preclinical stage of immune-mediated disease, but the onset of clinical disease may be postponed by active ovarian hormonal status.

Age adjusted and gender stratified model for men revealed that GADA (>5 U/ml) coexisted more likely with anti-TPO IgG low level (60–100 U/ml) when statistical analyses was controlled by age over 45 years (adOR = 12.18, *p* = 0.015). Similarly, GADA >30 U/ml coexisted more likely with anti-TPO IgG high level (>100 U/ml, adOR = 22.11, *p* = 0.003), when controlled by age. The presence of GADA >10 U/ml showed association with age over 45 years in men (adOR = 4.14, *p* = 0.010 when adjusted for the presence of anti-CTD IgG, and adOR = 3.82, *p* = 0.015 when adjusted for anti-TPO IgG). These results suggest that finding of anti-TPO IgG or GADA, regardless of level may reflect activation of adverse immune reaction which aggravates with age in men.

## Discussion

The data here represent a cross-sectional study of the prevalence of (auto)immune-mediated diseases in the general population of Estonia and the prevalence and association of 6 clinically significant autoantibodies in 994 (auto)immune-disease-free individuals from the general population. Most importantly, the prevalence of anti-TPO IgG and 5 tissue non-specific autoantibodies, anti-tTG IgG, and IgA, anti-CCP IgG, anti-CTD IgG, and GADA, in disease-free individuals was 23.6%. This was comparable to the prevalence of (auto)immune-mediated diseases registered in the general population of Estonia. In addition, several phenotypic parameters were associated with the presence of autoantibodies. These associations were gender specific and distinct for anti-TPO and tissue non-specific autoantibodies.

The surveying of a population-based registry of 51,862 individuals revealed that a third of the Estonian adult population had been diagnosed with some type of (auto)immune-mediated disease. The list of (auto)immune-mediated diseases was selected to include most diseases’ locations in the body and covered 90 ICD-10 categories (Supplementary Table S.1). Overall, men were diagnosed less frequently than women; 26.4% of men and 32.1% of women had at least one diagnosis (Table 1). Data indicating the prevalence of so many immune-mediated diseases in a population is rarely available in the scientific literature, which makes this study unique. According to one previous study, subjects with chronic diseases account for one-third of the general population in Germany^2^, although the list of studied diseases was different from that in this study.

The prevalence of disease was high, as was the prevalence of at least 1 of 6 autoantibodies in the population of disease-free individuals, especially among women (diseases occurred in 32.1% (31.6–32.6) of women in the general population and autoantibodies were present in 29.4% (25.5–33.6) of diseases-free women (proportion test, *p* > 0.05). Autoantibodies measured here in healthy individuals are presented with previously published data in Table 4. The prevalence of anti-tTG IgA, anti-CCP IgG, anti-CTD IgG and anti-TPO IgG in our study is comparable to the autoantibodies in general population of other countries^12^,^13^,^14^,^15^. The prevalence of GADA^16^,^17^ or autoantibodies from CTD IgG panel^17^,^18^,^19^,^20^ is not comparable due to differences either in laboratory tests or study populations.

Importantly, the autoantibodies detected in this study, anti-tTG IgG and IgA, anti-CTD IgG, GADA, and anti-TPO IgG, have diagnostic value for common autoimmune diseases, and at least some of these may play roles in disease pathogenesis. Although autoantibodies were assessed in healthy individuals, the positive cut-off value for each autoantibody test was chosen to match the level used to diagnose the corresponding autoimmune disease. Logically, it could be hypothesized that the group of seropositive but healthy individuals will develop autoimmune diseases, and the presence of autoantibodies is prognostic marker that precedes clinical manifestation. On the other hand, some of autoantibody-positive, disease-free individuals may never develop clinical disease, and the presence of autoantibodies may have no clinical significance for them. Although it is not known which of the above scenarios occurs, the current study provides important information. Long-term follow-up would be the best way to resolve this question.

In men, the presence of anti-TPO was strongly associated with familial anamnesis of maternal autoimmune disease. The same association was not revealed in women, although maternal autoimmune disease was reported more often in women than men (16.7% vs. 11.4%). Maternal thyroid disease, in particular, accounted for a negligible proportion of maternal autoimmune diseases in our cohort (reported in 4 cases, 1 woman and 3 men). Therefore, it seems likely that maternal autoimmunity, not specifically thyroid disease, leads to anti-thyroid autoimmunity in sons. This mother-to-son inheritance relies on several phenomena. First, the X-chromosome contains immunologically important genes, associated with a variety of autoimmune diseases^21^. Therefore, because male offspring inherit maternal X-chromosome, autoimmune susceptibility may be inherited if the mother is affected. Second, an increased frequency of skewed X-chromosome inactivation (XCI) has been found in many autoimmune diseases^22^ and may result from chance or genetic factors^23^. These sons may inherit the defective X-chromosome, which cannot be balanced because of the lack of spare X-chromosome in males, in contrast to the case in daughters. In this case, the mechanism of XCI to favour autoimmunity has been attributed to the potential escape of X-linked self-antigens in the thymus or other peripheral sites that are involved in tolerance^24^. However, these two explanations do not explain why the thyroid gland is preferentially targeted in sons of mothers suffering from autoimmune diseases. The third possible explanation involves maternal microchimerism, which has been detected in the newborn thyroid gland^25^. Regardless of the reason for anti-TPO antibodies in male participants, anti-TPO IgG can activate complement and cause damage to thyroid cells via antibody dependent cell cytotoxicity^13^ eventually leading to thyroid disease. Accordingly, men with anti-thyroid autoantibodies have a 5 times greater risk of progressing to overt thyroid gland disease than women^6^. Indeed, women may be protected from autoimmune diseases until menopause, because active ovarian hormones postpone the production of anti-TPO and tissue non-specific autoantibodies in women^26^.

Multivariate association analyses detected several phenotypic associations, suggesting the presence of at least one tissue non-specific autoantibody associated with moderate but regular alcohol intake and the need for cardiovascular treatment. These associations were independent of age and they were present in men but not women. The impacts of alcohol consumption on health are complex and modulated by several factors such as pattern and amount of drinking, genetics, the organ system studied, and the sex and age of the user^27^. Regular heavy consumption (≥3 drinks a day in men) causes suppression of innate immunity^28^, whereas moderate consumption (<2 drinks a day for men)^29^ may enhance the effects of vaccines^27^ and increase intestinal permeability^30^, which can lead to immune recognition of self-antigens^31^. Here, the effects of alcohol were not considered beneficial, since autoantibodies can mark future autoimmunity. Similarly, most of the benefits of moderate alcohol consumption have been described in the context of cardiovascular disease^32^. Here, we revealed that cardiovascular diseases were associated with increased prevalence of tissue non-specific autoantibodies, regardless of whether the person was consuming alcohol or not, which has been suggested previously^33^. Surprisingly, these associations were revealed in men and were not significant in women. Since women were tested for risk factors by statistical models adjusted for active ovarian hormones, other phenotypic parameters seem less important than ovarian hormonal profiles in women.

Childbearing age seemed to be the major protective factor from autoimmune disease in women. Indeed, women with active ovarian hormones (either because they were in their fertile years or receiving HRT)^12^ were less likely to be seropositive for anti-TPO and tissue non-specific autoantibodies regardless of confounders. Age related protection against autoimmunity can be explained by age related acquired X-chromosome loss or monosomy (XCM)^14^. Because the X-chromosome has immunologically important genes, XCM contributes to the speed of development and the number of overlapping autoimmune diseases^15^. In addition, the X-chromosome contains genes that appear to be crucial in the maintenance of physiological sex hormone levels^15^. Commonly, a substantial decline of sex hormone levels in women occurs at menopause. However, female sex hormones have various immunomodulatory effects^26^. These factors may explain why women were found to be likely autoantibody-positive in this study.

In conclusion, approximately one-third of the adult population of Estonia, individuals who lacked (auto)immune-mediated diseases, tested positive for at least one of six clinically significant autoantibodies. At the same time, the documented prevalence of the corresponding (auto)immune-mediated diseases in the general population was also one-third. However, some of these diseases, including thyroid disease and coeliac disease, were diagnosed less frequently than estimated. The results suggest that the presence of tissue non-specific autoantibodies may serve as prognostic markers for future diseases in currently disease-free individuals. The presence of autoantibodies in men depends on general health, age or health manners. In contrast, the protection of active ovarian hormones in women decrease any putative risk of health parameters studied here. Different from anti-TPO IgG in women, incidental finding of anti-TPO IgG in men with positive familial anamnesis of maternal autoimmune disease deserves further medical intention. The wider implications of these findings suggest that physicians should be encouraged to look for autoimmune markers in addition to treating a variety of patient health complaints.

## Additional Information

**How to cite this article:** Haller-Kikkatalo, K. *et al*. Demographic associations for autoantibodies in disease-free individuals of a European population. *Sci. Rep.* **7**, 44846; doi: 10.1038/srep44846 (2017).

**Publisher's note:** Springer Nature remains neutral with regard to jurisdictional claims in published maps and institutional affiliations.

## Supplementary Material

Supplementary Information

## Figures and Tables

**Table 1:** Prevalence of common (auto)immune-mediated diseases in Estonian adults.

Disease category	ICD-10^2^ category or subcategory	% (95% confidence interval)		
		Men N = 17,821	Women N = 34,041	Total N = 51,862
**Hypo-, hyperthyroidism or thyroiditis**	E03.4, E03.5, E03.8, E03.9, E05.0, E05.9, E06.2, E06.3	**0.5% (0.4–0.6)**	**3.1% (2.9–3.3)**	2.2% (2.1–2.3)
**Coeliac disease**	K90.0	0.04% (0.02–0.08)	0.05% (0.03–0.08)	0.04% (0.03–0.07)
**Rheumatoid arthritis**	M05.0, M05.1, M05.3, M05.4, M05.8, M05.9, M06.0, M06.1, M06.2, M06.3, M06.4, M06.8, M06.9	**0.6% (0.5–0.7)**	**1.9% (1.7–2.0)**	1.4% (1.3–1.5)
**Insulin-dependent (type 1) diabetes mellitus**	E10	**0.5% (0.4–0.6)**	**0.3% (0.2–0.3)**	0.4% (0.3–0.4)
**Other diabetes mellitus**	E11-14	**5.4% (5.1–5.8)**	**4.5% (4.3–4.7)**	4.8% (4.6–5.0)
**Diseases of the skin, subcutaneous tissue, musculoskeletal system and connective tissue**	L40, L63, L80, L93, M05-09, M12, M30-36, M45, M46	**5.3% (5.0–5.7)**	**7.1% (6.8–7.4)**	6.5% (6.3–6.7)
**Total**^1^		**26.4% (25.7–27.0)**	**32.1% (31.6–32.6)**	30.1% (29.7–30.5)

^1^Includes all immune mediated diseases listed in Supplementary Table S.1. The prevalence in each gender was compared with the Proportion test. Bold indicates *p* < 0.05.

^2^ICD-10 − International Classification of Diseases-10

**Table 2:** Reported phenotypic characteristics of the study population.

Characteristics^1^	Men (*n* = 491)	Women (*n* = 503)	Total (*n* = 994)
Age at time of study (yr)	39.8 ± 16.5	39.9 ± 16.1	39.9 ± 16.3
Place of birth			
City	302 (63.2%, 58.7–67.5)	310 (64.2%, 59.7–68.4)	612 (63.7%, 60.5–66.7)
Countryside	176 (36.8%, 32.5–41.3)	173 (35.8%, 31.6–40.3)	349 (36.3%, 33.3–39.5)
Occupation			
Employed	320 (66.8%, 62.4–71.0)	330 (68.3%, 63.9–72.4)	650 (67.6%, 64.5–70.5)
Pension insurance^2^	**43 (9.0%, 6.6–12.0)**	**28 (5.8%, 4.0–8.4)**	71 (7.4%, 5.8–9.3)
Student or serviceman	56 (11.7%, 9.0–15.0)	46 (9.5%, 7.1–12.6)	102 (10.6%, 8.8–12.8)
Unemployed^3^	**60 (12.5%, 9.8–15.9)**	**79 (16.4%, 13.2–20.0)**	139 (14.4%, 12.3–16.9)
Smoking status			
Never smoker	**230 (46.8%, 42.4–51.4)**	**312 (62.0%, 57.6–66.3)**	542 (54.5%, 51.4–57.6)
Former smoker	**89 (18.1%, 14.9–21.9)**	**50 (9.9%, 7.5–13.0)**	139 (14.0%, 11.9–16.3)
Current smoker	**172 (35.0%, 30.8–39.5)**	**142 (28.2%, 24.4–32.4)**	313 (31.5%, 28.6–34.5)
Frequency of alcohol consumption			
Rare – up to few times a year	**32 (9.9%, 7.0–13.8)**	**67 (21.3%, 17.0–26.3)**	99 (15.5%, 12.8–18.6)
Seldom – up to every month	**48 (14.8%, 11.2–19.3)**	**74 (23.5%, 19.0–28.6)**	122 (19.1%, 16.2–22.4)
Moderate or frequent – up to every 2nd day	**244 (75.3%, 70.2–79.8)**	**174 (55.2%, 49.6–60.8)**	418 (65.4%, 61.6–69.1)
Age when started smoking (yr)	**18.0 ± 4.0**	**20.6 ± 6.5**	19.7 ± 5.4
Has been pregnant	—	365 (72.6%, 68.4–76.4)	—
Age at first pregnancy (yr)	—	22.0 ± 3.8	—
Live birth(s)			
No children	—	138 (27.4%, 23.6–31.6)	—
1		97 (19.3%, 16.0–23.1)	
2	—	158 (31.4%, 27.4–35.7)	
≥3		89 (17.7%, 14.5–21.4)	
Active ovarian hormones^4^	—	349 (69.7%, 65.4–73.6)	—
Body mass index (kg/m^2^)			
Normal 18.5–24.9	**221 (45.0%, 40.6–49.5)**	**271 (54.0%, 49.5–58.4)**	492 (49.5%, 46.4–52.7)
Underweight <18.5	8 (1.6%, 0.8–3.3)	16 (3.2%, 1.9–5.2)	24 (2.4%, 1.6–3.6)
Overweight 25.0–29.9	**186 (37.9%, 33.6–42.4)**	**129 (25.7%, 22.0–29.8)**	315 (31.7%, 28.9–34.7)
Obesity ≥30	76 (15.5%, 12.5–19.1)	86 (17.1%, 14.0–20.8)	162 (16.3%, 14.1–18.8)
Recently used drugs^5^			
Cardiovascular system (C)	82 (16.7%, 13.6–20.4)	77 (15.3%, 12.3–18.8)	159 (16.0%, 13.8–18.5)
Urogenital tract (G)	6 (1.2%, 0.5–2.8)	7 (1.4%, 0.6–3.0)	13 (1.3%, 0.7–2.3)
Central nervous system (N)	41 (8.4%, 6.1–11.2)	40 (8.0%, 5.8–10.8)	81 (8.1%, 6.6–10.1)
Respiratory tract (R)	14 (2.9%, 1.6–4.9)	11 (2.3%, 1.1–4.0)	25 (2.5%, 1.7–3.7)
Immune system (H, J, L)	16 (3.3%, 1.9–5.4)	25 (5.0%, 3.3–7.4)	41 (4.1%, 3.0–5.6)
Subgroup of immunity-affecting drugs^6^	68 (13.8%, 11.0–17.3)	78 (15.5%, 12.5–19.0)	146 (14.7%, 12.6–17.1)
Maternal autoimmune disease^7^	**56 (11.4%, 8.8**–**14.6)**	**84 (16.7%, 13.6**–**20.3)**	140 (14.1%, 12.0–16.4)
Paternal autoimmune disease^8^	**62 (12.6%, 9.9**–**16.0)**	**39 (7.8%, 5.6**–**10.5)**	101 (10.2%, 8.4–12.2)

^1^Participants lacking data were excluded from this data calculation.

^2^Pension insurance includes retired persons and unemployed persons with insurance for disability.

^3^Unemployed includes women on childcare leave.

^4^Women with active ovarian hormones includes women who menstruate and women with primary or secondary amenorrhea who are currently receiving hormone replacement therapy.

^5^Drugs used regularly during last 2 months were classified according to the Anatomical Therapeutic Chemical Classification System.

^6^Drugs that increase risk of developing lupus or other autoimmune diseases^10^.

^7^Participants who reported that their mother had at least one of the following autoimmune diseases (ICD-10): thyroiditis (E06), insulin dependent diabetes (E10), other adrenal diseases (E27), sclerosis multiplex (G35), sleep disorders (G47), rhinitis (J30), asthma (J45), atopic dermatitis (L20), psoriasis (L40), urticaria (L50), vitiligo (L80), arthropathies (M00-25), seropositive rheumatoid arthritis (M05), other rheumatoid arthritis (M06), or systemic sclerosis (M34).

^8^Participants who reported that their father had at least one of the following autoimmune diseases (ICD-10): vitamin B12 deficiency (D51), thyrotoxicosis (E05), insulin-dependent diabetes (E10), sclerosis multiplex (G35), sleep disorders (G47), rhinitis (J30), asthma (J45), atopic dermatitis (L20), allergic contact dermatitis (23), psoriasis (L40), seropositive rheumatoid arthritis (M05), other rheumatoid arthritis (M06), systemic sclerosis (M34), other systemic involvement of connective tissue (M35), ankylosing spondylitis (M45). Statistically significant differences (*p* < 0.05) are shown in bold.

Numeric data are provided as means ± standard deviation and were compared between men and women with *t*-test. Non-parametric data are provided as count; the percentage of a row and 95% confidence intervals of percentage were compared between genders with a proportion test.

**Table 3:** Prevalence of autoantibodies in the study population.

Autoantibodies	Population	% (95% confidence intervals)		
		Men	Women	Total
		*n* = 491	*n* = 503	*n* = 994
(1) Anti-tTG IgA	994	**0.0**	**1.2 (0.5–2.7)**	0.6 (0.2–1.4)
(2) Anti-tTG IgG	994	**0.0**	**0.4 (0.1–1.6)**	0.2 (0.0–0.8)
(3) Anti-CCP IgG	994	**0.0**	**1.0 (0.4–2.4)**	0.5 (0.2–1.2)
(4) Anti-CTD IgG test	994	**3.1 (1.8–5.1)**	**6.6 (4.6–9.2)**	4.8 (3.6–6.4)
Anti-dsDNA IgG	851	1.0 (0.4–2.5)	2.2 (1.2–4.0)	1.6 (1.0–2.7)
Anti-SS-B/La IgG	85^1^	**0.2 (0.0–1.3)**	**0.0**	0.1 (0.0–0.7)
Anti-SS-A/Ro IgG	85^1^	0.4 (0.1–1.6)	1.0 (0.4–2.4)	0.7 (0.3–1.5)
Anti-Sm IgG	85^1^	0.0	0.0	0.0
Anti-U1RNP IgG	85^1^	0.0	0.2 (0.0–1.3)	0.1 (0.0–0.7)
Anti-CENP IgG	85^1^	**0.0**	**0.6 (0.2–1.9)**	0.3 (0.1–1.0)
Anti-Jo-1 IgG	85^1^	0.0	0.0	0.0
Anti-Scl-70 IgG	85^1^	0.0	0.0	0.0
(5) GADA Ig^2^	994	7.9 (5.8–10.8)	9.5 (7.2–12.5)	8.8 (7.1–10.7)
≥1 tissue non-specific autoantibodies^3^	994	**14.7 (11.7**–**18.2)**	**19.9 (16.5**–**23.7)**	17.3 (15.0–19.8)
(6) Anti-TPO IgG	994	**3.7 (2.3–5.8)**	**10.7 (8.2–13.9)**	7.2 (5.7–9.1)
≥1 any autoantibodies^4^	994	**17.7 (14.5**–**21.5)**	**29.4 (25.5**–**33.6)**	23.6 (21.1–26.4)

^1^Antigen-specifying tests were performed on participants who were borderline positive for antinuclear autoantibodies, and the test results (prevalence) were expanded to entire study population.

^2^Ca^2+^-treated peripheral blood plasma.

^3^Presence of at least one of five tissue non-specific autoantibodies.

^4^Presence of at least one of six measured autoantibodies.

Data are provided as percentage and 95% CI of percentage and prevalence was compared between genders with a proportion test. CCP - cyclic citrullinated peptide, CENP - centromere protein, dsDNA – double-stranded deoxyribonucleic acid, GADA - autoantibodies against glutamic acid decarboxylase (molecular weight of 65 kDa), Jo-1 – histidyl-tRNA synthetase, SS-A/Ro - Sjögren’s syndrome type A antigen, SS-B/La - Sjögren’s syndrome type B antigen, Scl-70 - scleroderma-associated autoantigen of 70k Da, Sm - Smith protein, TPO – thyroid peroxidase, tTG - tissue transglutaminase, U1RNP - ribonucleoprotein U1. Statistically significant differences (*p* < 0.05) are shown in bold.

**Table 4:** Extended commented presentation of autoantibody prevalence of current study and from literature data.

Autoantibody				Corresponding disease and its prevalence % (95% CI)	Comments
Name	Prevalence % (95% CI)	Population	Method		
Anti-tTG IgA	0.6% (0.2–1.4)	Immune-mediated disease-free adults, Estonia	FEIA*	*Coeliac disease:* Reported in 0.04% (0.03–0.07) of general population, Estonia^34^, Estimated in 0.5–1.0% of general population, USA, UK, and other European countries (for review see ref. 35). Estimated in 0.5% (0.2–0.8) of general population of adults, Denmark^36^	According to the prevalence and specificity of anti-tTG IgA^37^,^38^, coeliac disease is clearly underdiagnosed at least in Estonia.
	0.3% (0.1–0.9)	School children, Estonia	FEIA^34^		
	0.8%	General population, Natrona County, USA	ELISA^39^		
Anti-CCP IgG	0.5% (0.2–1.2)	Immune-mediated disease-free adults, Estonia	FEIA*	*Rheumatoid arthritis*:Reported in 1.4% (1.3–1.5) of general population, Estonia,^34^ *Systemic sclerosis*: Estimated in 0.024% of general population, Australia, Spain, USA (for review see ref. 40)	Logical difference between autoantibody-prevalence in disease-free individuals and two times high prevalence in general population. Autoantibody prevalence is comparable to reported cases of rheumatoid arthritis in general population.
	1% (0-5)	General population, Salt Lake City, Utah, USA	ELISA^41^		
	1%	General population, Southern Brazil	*NA*^42^		
	65% (56–73)	Patients with rheumatoid arthritis, Salt Lake City, Utah, USA	ELISA^41^		
	11.5%	Patients with systemic sclerosis, Southern Brazil	*NA*^42^		
Anti-CTD IgG:	4.8% (3.6–6.4)	Immune-mediated disease-free adults, Estonia	FEIA*	*Various CTDs:* Reported diseases of the skin, subcutaneous tissue, musculoskeletal system and connective tissue in 6.5% (6.3–6.7) of general population, Estonia^34^	Antinuclear autoantibodies detected by CTD panel have similar prevalence in different populations and reflect general prevalence of CTDs.
	<4%	General population, Belgium	FEIA^20^		
	2.0%	General population, Australia	ELISA^19^		
Anti-dsDNA	1.6%(1.0–2.7)	Immune-mediated disease-free adults, Estonia	FEIA*	*Lupus erythematosus*^43^: Reported in 0.06% (0.05–0.07) in general population, Buenos Aires, Argentina^44^	Specified panel of anti-CTD has been provided in diseased persons, mostly with systemic lupus erythematosus or has been detected by other than FEIA^45^,^46^. Also, since particular ANA are not specific to just one disease, the prevalence of ANAs in disease-free individuals and particular CTD in general population relate reasonably.
	33.7%	Patients with lupus erythematosus, Brazil	*NA*^47^		
Anti-SS-B/La	0.1% (0.0–0.7)	Immune-mediated disease-free adults, Estonia	FEIA*	*Mild Sjögren´s syndrome*^43^	
	36%	Primary Sjögren´s syndrome, Italy	ELISA^48^		
Anti-SS-A/Ro	0.7% (0.3–1.5)	Immune-mediated disease-free adults, Estonia	FEIA*	*Vasculitis and nephritis of CTD, Sjögren´s syndrome*^43^	
	1.9% (0–6.0)	General population, Denmark	IIF^49^		
	68%	Patients with primary Sjögren´s syndrome, Italy	ELISA^48^		
	SS-A/SS-B 16.5%	Disease-free individuals with HLA-DR3 or/and DR11 allele and autoantibodies to enteroviruses, Estonia	ELISA^18^		
Anti-Sm	0.0%	Immune-mediated disease-free adults, Estonia	FEIA*	*Lupus erythematosus*^50^	
	0–45%	Patients with lupus erythematosus, Hong Kong, China	ELISA^50^		
Anti-U1RNP	0.1% (0.0–0.7)	Immune-mediated disease-free adults, Estonia	FEIA*	*Mixed connective tissue disease and mild Sjögren´s syndrome*^43^	
	0–84%	Patients with lupus erythematosus, Hong Kong, China	ELISA^50^		
Anti-CENP	0.3% (0.1–1.0)	Immune-mediated disease-free adults, Estonia	FEIA*	*CREST (calcinosis, Raynaud’s, oesophageal dysmotility, sclerodactyly, and telangiectasia) syndrome* 43	
Anti-Jo-1	0%	Immune-mediated disease-free adults, Estonia	FEIA*	*Pulmonary fibrosis and poor prognosis of CTD*^43^	
Anti-Scl-70	0%	Immune-mediated disease-free adults, Estonia	FEIA*	*Pulmonary fibrosis and poor prognosis of CTD*^43^	
GADA	8.8% (7.1–10.7) ≥5 U/ml	Immune-mediated disease-free adults, Estonia	Ca-treated plasma ELISA*	*Insulin dependent diabetes mellitus:* Reported in 0.4% (0.3–0.4) of general population, Estonia^17^ Reported and estimated 1.1–9% in general population, Europe^51^*Type 1 diabetes:*Estimated in 0.6–0.8% of general population, Europe^51^ *Latent autoimmune diabetes of adults:* Estimated in 0.5–0.7% of general population, Europe^52^	GADA in disease-free individuals reflect the prevalence of autoimmune diabetes in general population. Calcium treatment of plasma has been suggested prior to detection GADA from EDTA-plasma^11^, but may still be unsuitable for detecting GADA by ELISA. Alternatively, higher cut-off values may suit for GADA ELISA from plasma, leaving out GADA with low affinity and insignificance^53^.
	3.4% (2.4–4.8) ≥10 U/ml	Immune-mediated disease-free adults, Estonia	Ca-treated plasma ELISA*		
	0.4% (0.1–1.1) ≥50 U/ml	Immune-mediated disease-free adults, Estonia	Ca-treated plasma ELISA*		
	0.9%	General population, Southern Spain	*NA*^16^		
	2.0% (0.6-5.4)	Disease-free adults from rural town, Estonia	Serum RIA^17^		
Anti-TPO IgG	7.2% (5.7–9.1)	Immune-mediated disease-free individuals, Estonia	FEIA*	*Hypo- and hyperthyroidism*: Reported in 2.2% (2.1–2.3) of general population, Estonia* and in 5% of women population, California, USA^54^ *Hypothyroidism:* Screened in 12% of complaint-free individuals, but reported in <2% of general population, Michigan, USA^55^	Anti-TPO prevalence in disease-free individuals seems to be comparable to the estimated prevalence of thyroid diseases in general population but higher than reported cases. Thyroid diseases are underdiagnosed.
	3.3%	Disease-free children and adolescents, Leipzig, Germany	ECLIA^56^		
	10% (5–15)	General population, Denmark	Plasma RIA^49^		
	8.6%	General population, Australia	ELISA^19^		
	10.7%(8.2–13.9)	Immune-mediated disease-free women, Estonia	FEIA*		
	17%	General population, Iran	Serum RIA^57^		
Co-existed anti-TPO and GADA	0.6% (0.2–1.4)	Immune-mediated disease-free adults, Estonia	FEIA and ELISA*	*Thyrogastric autoimmune disease*^58^: 17.8% of type 1 diabetes patients had anti-TPO, Turkey^59^	Taken the prevalence of type 1 diabetes^51^, the calculated co-existence of anti-TPO and GADA in Turkey^59^ is lower than detected among disease-free individuals in Estonia*. The prevalence of co-existence of these autoantibodies is likely population-specific.
	0.11–0.14%	Patients with type 1 diabetes and anti-TPO, Turkey	ELISA^59^		

ANA – antinuclear autoantibodies, ACLIA – electroluminescence assay, ELISA – enzyme linked immunosorbent assay, CTD – connective tissue diseases, CCP - cyclic citrullinated peptide, CENP - centromere protein, dsDNA - double stranded deoxyribonucleic acid, FEIA - fluoro-enzyme immunoassay, GADA - autoantibodies against glutamic acid decarboxylase (molecular weight 65 kDa), IIF – indirect immunofluorescent test, Jo-1 - histidyl tRNA synthetize, NA – not available, RIA – radioimmunoassay, SS-A/Ro - Sjögren’s syndrome type A antigen, SS-B/La - Sjögren’s syndrome type B antigen, Scl-70 - scleroderma-associated autoantigen of 70 kDa, Sm - Smith protein, TPO – thyroid peroxidase, tTG - tissue transglutaminase, U1RNP - ribonucleoprotein U1. *Current article by Haller-Kikkatalo K., Alnek K., Metsküla K., Kisand K., Pisarev H., Salumets A., Uibo R.

**Table 5:** Associations between autoantibodies and phenotypic characteristics.

Phenotypic characteristics^1^	Association with phenotypic characteristics			
	≥1 tissue non-specific autoantibody^6^		Anti-TPO	
	Men	Women	Men	Women
Age at time of study (years)	OR = 1.03*	OR = 1.01	OR = 1.02	OR = 1.03*
Age groups^2^				
18–26 years	OR = 1	OR = 1	OR = 1	OR = 1
27–37 years	OR = 2.08#	OR = 0.86	OR = 0.67	OR = 1.76
38–52 years	OR = 2.47*	OR = 1.21	OR = 1.69	OR = 1.84
>52 years	OR = 3.39*	OR = 1.40	OR = 1.58	OR = 3.88*
Occupation				
Employed	adOR = 1	adOR = 1	adOR = 1	adOR = 1
Pension insurance^3^	adOR = 2.48	adOR = 0.82	adOR = 0.49	adOR = 2.19
Student or serviceman	adOR = 0.98	adOR = 1.56	adOR = 3.72#	adOR = 1.08
Unemployed^4^	adOR = 1.64	adOR = 1.10	adOR = 0.46	adOR = 0.99
Frequency of alcohol consumption				
No consumption	adOR = 1	adOR = 1	adOR = 1	adOR = 1
Rare – up to few times per year	adOR = 1.74	adOR = 1.31	adOR = 0.62	adOR = 0.81
Seldom – up to every month	adOR = 2.92*	adOR = 1.44	adOR = 2.05	adOR = 1.75
Moderate or frequent – up to every 2^nd^ day	adOR = 1.45	adOR = 0.93	adOR = 1.10	adOR = 0.69
Active ovarian hormones^5^	—	adOR = 0.50*	—	adOR = 0.55
Recently used cardiovascular drugs	adOR= 1.85#	adOR = 1.19	adOR = 1.12	adOR = 0.85
Maternal autoimmune disease	adOR = 0.38	adOR = 1.11	adOR = 5.50*	adOR = 1.17

^1^Characteristics with any association with either anti-TPO or tissue non-specific autoantibodies are presented.

^2^Age groups were formed according to the values of the 1^st^ and 3^rd^ quantiles and median of the age and compared with the youngest group (18-26 years).

^3^Pension insurance includes retired persons and individuals who are unemployed but covered by health insurance.

^4^Unemployed also includes women on childcare leave.

^5^Women with active ovarian hormones includes women who menstruate and women with primary or secondary amenorrhea who are currently receiving hormone replacement therapy.

^6^The presence of at least one of five tissue non-specific autoantibodies (antinuclear autoantibodies detected with a connective tissue disease screening test and autoantibodies against glutamic acid decarboxylase (molecular weight of 65 kDa), thyroid peroxidase, tissue transglutaminase IgG and IgA, or cyclic citrullinated peptide). adOR – adjusted odds ratio.

*Statistically significant association (p < 0.05), # statistical tendency towards association (0.05 < p < 0.1). Associations between phenotypic characteristics and autoantibody prevalence were analysed with logistic regression analyses stratified by gender and adjusted for age.

**Table 6:** GADA autoantibodies of different level.

GADA cut-off level	GADA positive individuals (%, 95% confidential interval)	
	Men (N = 491)	Women (N = 503)
>5 U/ml	39/491 (7.9%, 5.8–10.8)	48/503 (9.5%, 7.2–12.5)
<45 years	19/39 (48.7%, 32.7–65.0)	23/48 (47.9%, 33.5–62.6)
>45 years	20/39 (51.3, 35.0–67.3)	25/48 (52.1%, 37.4–66.5)
Active ovarian hormones		21/48 (43.8%, 29.8–58.7)
Non-active ovarian hormones		26/48 (54.2%, 39.3–68.4)
>10 U/ml	16/491 (3.3%, 1.9–5.4)	18/503 (3.6%, 2.2–5.7)
<45 years	5/16 (31.3%, 12.1–58.5)*	8/18 (44.4%, 22.4–68.7)
>45 years	11/16 (68.8%, 41.5–87.9)*	10/18 (55.6%, 31.3–77.6)
Active ovarian hormones		8/18 (44.4%, 22.4–68.7)
Non-active ovarian hormones		10/18 (55.6%, 31.3–77.6)
>30 U/ml	4/491 (0.8%, 0.3–2.2)	7/503 (1.4%, 0.6–3.0)
<45 years	1/4 (25.0%, 1.3–78.1)	3/7 (42.9%, 11.8–79.8)
>45 years	3/4 (75.0%, 21.9–98.7)	4/7 (57.1%, 20.2–88.2)
Active ovarian hormones		2/7 (28.6%, 5.1–69.7)*
Non-active ovarian hormones		5/7 (71.4%, 30.3–94.9)*
>50 U/ml	1/491 (0.2%, 0–1.3)	3/503 (0.6%, 0.2–1.9)
<45 years	0/1 (0%, 0–94.5)*	1/3 (33.3%, 1.8–87.5)
>45 years	1/1 (100%, 5.4–100)*	2/3 (66.7%, 12.5–98.2)
Active ovarian hormones		1/3 (33.3%, 1.8–87.5)
Non-active ovarian hormones		2/3 (66.7%, 12.5–98.2)

GADA prevalence are provided among study population and not extended to entire Estonian population (compare Table 3 and 4). *proportion test p < 0.05 between rows.
