# Supplementary material for: Demographic associations for autoantibodies in disease-free individuals of a European population
Source: Sci Rep. 2017 Mar 28;7:44846. doi: 10.1038/srep44846 (PMC5368634; doi:10.1038/srep44846)
Supplement: Supplementary Information [file srep44846-s1.pdf]

Demographic associations for autoantibodies in disease-free individuals of a European population

Kadri Haller-Kikkatalo 1, 2, 3, *, Kristi Alnek 1, *, Andres Metspalu 4, 5, Evelin Mihailov 4, Kaja Metsküla 1, Kalle Kisand 1, Heti Pisarev 6, Andres Salumets 2, 3, 7, 8, Raivo Uibo 1, 2

1 Institute of Bio- and Translational Medicine, Department of Immunology, University of Tartu, Ravila 19, Tartu 50411, Estonia

2 Competence Center on Health Technologies, Tiigi 61b, Tartu 50410, Estonia

3 Institute of Clinical Medicine, Department of Obstetrics and Gynecology, University of Tartu, L. Puusepa 8, Tartu 51014, Estonia

4 Estonian Genome Center, University of Tartu, Riia 23b, Tartu 51010, Estonia

5 Institute of Molecular and Cell biology, University of Tartu, Riia 23, Tartu 51010

6 Department of Public Health, University of Tartu, Ravila 19, Tartu 50411, Estonia

7 Institute of Bio- and Translational Medicine, Department of Biomedicine, University of Tartu, Ravila 19, Tartu 50411, Estonia

8 Department of Obstetrics and Gynecology, University of Helsinki and Helsinki University Hospital, Helsinki, FI-00029 HUS, Finland

* Contributed equally

Corresponding author: Kadri Haller-Kikkatalo, kadri.haller-kikkatalo@ut.ee, Phone: +372-50-20027, Fax: +372-7-7374232

Supplementary Table S. 1. Exclusion criteria according to the International Statistical Classification of Diseases (ICD-10).

ICD category	Disease	ICD subcategory
B20	Human immunodeficiency virus (HIV) disease resulting in infectious and parasitic diseases	B20.0, B20.1, B20.2, B20.3, B20.4, B20.5, B20.6, B20.7, B20.8, B20.9
B21	Human immunodeficiency virus (HIV) disease resulting in malignant neoplasms	B21.0, B21.1, B21.2, B21.3, B21.7, B21.8, B21.9
B22	Human immunodeficiency virus (HIV) disease resulting in other specified diseases	B22.0, B22.1, B22.2, B22.7
B23	Human immunodeficiency virus (HIV) disease resulting in other conditions	B23.0, B23.1, B23.2, B23.8
B24	Unspecified human immunodeficiency virus (HIV) disease	
D47	Other neoplasms of uncertain or unknown behavior of lymphoid, hematopoietic and related tissue	D47.2
D77	Other disorders of blood and blood-forming organs in diseases classified elsewhere	D77.1
D80	Immunodeficiency with predominantly antibody defects	D80.0, D80.1, D80.2, D80.3, D80.4, D80.5, D80.6, D80.7, D80.8, D80.9
D81	Combined immunodeficiencies	D81.0, D81.1, D81.2, D81.3, D81.4, D81.5, D81.6, D81.7, D81.8, D81.9
D82	Immunodeficiency associated with other major defects	D82.0, D82.1, D82.2, D82.3, D82.4, D82.8, D82.9
D83	Common variable immunodeficiency	D83.0, D83.1, D83.2, D83.8, D83.9
D84	Other immunodeficiencies	D84.0, D84.1, D84.8, D84.9
D86	Sarcoidosis	D86.0, D86.1, D86.2, D86.3, D86.8, D86.9
D89	Other disorders involving the immune mechanism, not elsewhere classified	D89.0, D89.1, D89.2, D89.8, D89.9
E03	Other hypothyroidism	E03.4, E03.5, E03.8, E03.9
E05	Thyrotoxicosis (hyperthyroidism)	E05.0, E05.9
E06	Thyroiditis	E06.2, E06.3
E10	Insulin-dependent diabetes mellitus	E10.0, E10.1, E10.2, E10.3, E10.4, E10.5, E10.6, E10.7, E10.8, E10.9
E11	Non-insulin-dependent diabetes mellitus	E11.0, E11.1, E11.2, E11.3, E11.4, E11.5, E11.6, E11.7, E11.8, E11.9
E12	Malnutrition-related diabetes mellitus	E12.0, E12.1, E12.2, E12.3, E12.4, E12.5, E12.6, E12.7, E12.8, E12.9
E13	Other specified diabetes mellitus	E13.0, E13.1, E13.2, E13.3, E13.4, E13.5, E13.6, E13.7, E13.8, E13.9
E14	Unspecified diabetes mellitus	E14.0, E14.1, E14.2, E14.3, E14.4, E14.5, E14.6, E14.7, E14.8, E14.9
E27	Other disorders of adrenal gland	E27.1, E27.2i
E28	Ovarian dysfunction	E28.3

E31	Polyglandular dysfunction	E31.0
E66	Obesity	E66.0, E66.1, E66.2, E66.8, E66.9
E85	Amyloidosis	
E88	Other metabolic disorders	E88.0
G35	Multiple sclerosis	
G36	Other acute disseminated demyelination	G36.0, G36.1, G36.8, G36.9
G37	Other demyelinating diseases of central nervous system	G37.0, G37.1, G37.2, G37.3, G37.4, G37.5, G37.8, G37.9
G61	Inflammatory polyneuropathy	G61.0, G61.1, G61.8, G61.9
G63	Polyneuropathy in diseases classified elsewhere	G63.1, G63.5
G70	Myasthenia gravis and other myoneural disorders	G70.0, G70.2, G70.8, G70.9
G72	Other myopathies	G72.4
G73	Disorders of myoneural junction and muscle in diseases classified elsewhere	G73.0, G73.1, G73.2, G73.3
K50	Crohn's disease (regional enteritis)	
K51	Ulcerative colitis	
L10	Pemphigus	L10.0, L10.1, L10.2, L10.3, L10.4, L10.5, L10.8, L10.9
L12	Pemphigoid	L12.0, L12.1, L12.2, L12.3, L12.8, L12.9
L13	Other bullous disorders	L13.0, L13.1, L13.8, L13.9
L20	Atopic dermatitis	L20.0, L20.8, L20.9
L23	Allergic contact dermatitis	L23.0, L23.1, L23.2, L23.3, L23.4, L23.5, L23.6, L23.7, L23.8, L23.9
L24	Irritant contact dermatitis	
L25	Unspecified contact dermatitis	
L26	Exfoliative dermatitis	
L27	Dermatitis due to substances taken internally	
L28	Lichen simplex chronicus and prurigo	
L30	Other dermatitis	
L40	Psoriasis	L40.0, L40.1, L40.2, L40.3, L40.4, L40.5, L40.8, L40.9
L41	Parapsoriasis	L41.0, L41.1, L41.2, L41.3, L41.4, L41.5, L41.8, L41.9
L43	Lichen planus	L43.0, L43.1, L43.2, L43.3, L43.8, L43.9
L50	Urticaria	L50.0, L50.1, L50.9
L63	Alopecia areata	L63.0, L63.1, L63.2, L63.8, L63.9
L66	Cicatricial alopecia	L66.0, L66.1, L66.2, L66.3, L66.4, L66.8, L66.9
L80	Vitiligo	
L88	Pyoderma gangrenosum	
L92	Granulomatous disorders of skin and subcutaneous tissue	L92.0, L92.1, L92.2, L92.3, L92.8, L92.9
L93	Lupus erythematosus	L93.0, L93.1, L93.2

L94	Other localized connective tissue disorders	L94.0, L94.1, L94.2, L94.3, L94.4, L94.5, L94.6, L94.7, L94.8, L94.9
L95	Vasculitis limited to skin, not elsewhere classified	
L99	Other disorders of skin and subcutaneous tissue in diseases classified elsewhere	L99.0
M05	Seropositive rheumatoid arthritis	M05.0, M05.1, M05.3, M05.4, M05.8, M05.9
M06	Other rheumatoid arthritis	M06.0, M06.1, M06.2, M06.3, M06.4, M06.8, M06.9
M07	Psoriatic and enteropathic arthropathies	M07.0, M07.1, M07.2, M07.3, M07.4, M07.5, M07.6
M08	Juvenile arthritis	M08.0, M08.1, M08.2, M08.3, M08.4, M08.8, M08.9
M09	Juvenile arthritis in diseases classified elsewhere	M09.0, M09.1, M09.2, M09.8
M12	Other specific arthropathies	
M13	Other arthritis	
M30	Polyarteritis nodosa and related conditions	M30.0, M30.1, M30.2, M30.3, M30.8
M31	Other necrotizing vasculopathies	M31.0, M31.1, M31.2, M31.3, M31.4, M31.5, M31.6, M31.8, M31.9
M32	Systemic lupus erythematosus	M32.0, M32.1, M32.8, M32.9
M33	Dermatopolymyositis	M33.0, M33.1, M33.2, M33.9
M34	Systemic sclerosis	M34.0, M34.1, M34.2, M34.8, M34.9
M35	Other systemic involvement of connective tissue	M35.0, M35.1, M35.2, M35.3, M35.4, M35.5, M35.6, M35.7, M35.8, M35.9
M36	Systemic disorders of connective tissue in diseases classified elsewhere	
M45	Ankylosing spondylitis	
M46	Other inflammatory spondylopathies	
I00	Rheumatic fever without mention of heart involvement	
I01	Rheumatic fever with heart involvement	I01.0, I01.1, I01.2, I01.8, I01.9
I02	Rheumatic chorea	I02.0, I02.9
I05	Rheumatic mitral valve diseases	I05.0, I05.1, I05.2, I05.8, I05.9
I06	Rheumatic aortic valve diseases	I06.0, I06.1, I06.2, I06.8, I06.9
I07	Rheumatic tricuspid valve diseases	I07.0, I07.1, I07.2, I07.8, I07.9
I08	Multiple valve diseases	I08.0, I08.1, I08.2, I08.3, I08.8, I08.9
I09	Other rheumatic heart diseases	I09.0, I09.1, I09.2, I09.8, I09.9
O24	Diabetes mellitus in pregnancy	O24.0, O24.1, O24.2, O24.3, O24.4, O24.9
R73	Elevated blood glucose level	R73.0, R73.9
Q87	Other specified congenital malformation syndromes affecting multiple systems	Q87.1, Q87.8
Q90	Down's syndrome	Q90.0, Q90.1, Q90.2, Q90.9

Q96	Turner's syndrome	Q96.0, Q96.1, Q96.2, Q96.3, Q96.4, Q96.8, Q96.9
Q98	Other sex chromosome abnormalities, male phenotype, not elsewhere classified	Q98.0, Q98.1, Q98.2
T78	Adverse effects, not elsewhere classified (effects of external cause)	
T80	Complications following infusion, transfusion and therapeutic injection	
